# True vertical validation in facial orthognathic surgery planning

**DOI:** 10.4317/jced.51188

**Published:** 2013-12-01

**Authors:** Eduardo Espinar-Escalona, Maria B. Ruiz-Navarro, José M. Barrera-Mora, José M. Llamas-Carreras, Andreu Puigdollers-Pérez, Jorge Ayala-Puente

**Affiliations:** 1 Associate Professor of Orthodontics. School of Dentistry. University of Seville; 2 Master of Orthodontics and Dentofacial Orthopedics. University of Seville; 3 Professor of Orthodontics. School of Dentistry. International University of Cataluña (UiC); 4 Orthodontics. Santiago, Chile

## Abstract

Objectives: To validate the effectiveness of the original standards of True Vertical (TV) Subnasal Line in orthognatic surgery planning. The present study evaluates the changes occurring in patients with skeletal Class II alterations programmed for orthognathic surgery with a view to improving their facial profile. Study design: We showed a series of black profiles (composed by a first control group of subjects with normal occlusion, and another two additional groups comprised patients before –Group 2- and after orthognatic surgical correction of Class II malocclusion -Group 3-) for three groups of observers (orthodontists, surgeons and laypeople). The facial images became black silhouettes in order to determine a series of parameters (including aesthetic assessment) by means of the observers. Their observation were assessed using a 5-point Likert scale. Results: The sample was composed of 52 profile’s subjects who were tested for a total of 72 observers. Aesthetic assessment yielded mean scores of 2.57, 1.67 and 2.46 for groups 1, 2 and 3, respectively. There was a statistically significant difference (p<0.001) between group 1 versus group 2. There were no significant differences in terms of observer assessment of aesthetics, with the exception of a wider perception range among the orthodontists. Regarding the studied profile measures, significant differences were recorded for point B’ and Pg’ (p<0.02) between groups 2 and 3 (i.e., pre- versus post-surgery). Conclusions: The results of our study suggest the subnasale vertical and sagittal measures of the lower third of the face are decisive in facial aesthetics, and therefore also for the planning of orthognathic surgery. Consequently, these aesthetic parameters can be used as an objective tool for the planning of orthodontic treatment.

** Key words:**Facial profile, Class II, orthognathic surgery, cephalometric analysis, facial soft tissue, subnasale vertical.

## Introduccion

From the beginnings of Orthodontics, specialists in the field have been interested in the changes occurring in patient facial profile as a result of dental movements. These changes are particularly relevant in the case of patients programmed for orthognathic surgery. In individuals with severe dentoskeletal alterations in Class II malocclusions, mandibular advancement surgery offers improvement in the correction of malocclusion and in terms of the facial profile. In this context, Profitt (1) reported improved aesthetics in most patients subjected to orthognathic surgery with mandibular advancement, and particularly in those presenting a very low initial aesthetic assessment score.

In 1952, Herzberg (2) described the profiles of three individuals which he considered to be “harmonic”. Based on photographic analyses, this author established that the chin, upper lip and lower lip fell on a vertical line through the subnasion or subnasale. However, Herzberg made no mention of any kind of horizontal planes or vertical reference for constructing the reference line in the photographs.

Stoner (3), and later Peck and Peck (4), in turn studied aesthetically acceptable profiles based on photographs and using a vertical plane tangential to the soft nasion and pogonion. From this plane they established the sagittal positions for the upper and lower lip, and chin, with a base of angular measures. Holdaway (5) determined an angle of reference from the soft subnasale to the soft supra-pogonion and the Frankfort plane, corresponding to values taken to represent harmony of the facial profile. Merrifield (6) similarly analyzed the profiles with a line tangential to the soft pogonion and the most prominent lip, extended superiorly until intercepting the horizontal Frankfort plane. The inferoposterior angle formed by the intersection of both lines was referred to as the “Z angle”, the value of which offers some indication of the sagittal position of the lips and chin. Burstone (7) used a plane through the subnasale and tangential to the soft pogonion, establishing that this plane experiences minimum variations in patients out of growth. This author defined linear measures perpendicular to this plane for determining the normal positions of the most prominent points of the upper and lower lip.

González-Ulloa and Stevens (8) established a vertical plane through the soft nasion and perpendicular to the horizontal Frankfort plane. They found that in profiles considered to be attractive, the soft chin fell on this vertical plane.

Jacobson (9) used a true extracranial vertical as reference plane, obtained in the natural position of the head. This approach has been established as the most accurate method for obtaining a lateral X-ray of the skull. However, this author studied mainly sagittal discrepancies of the maxillas, not the linear or angular relationships of the soft tissues in relation to the true vertical.

**Figure 1:**
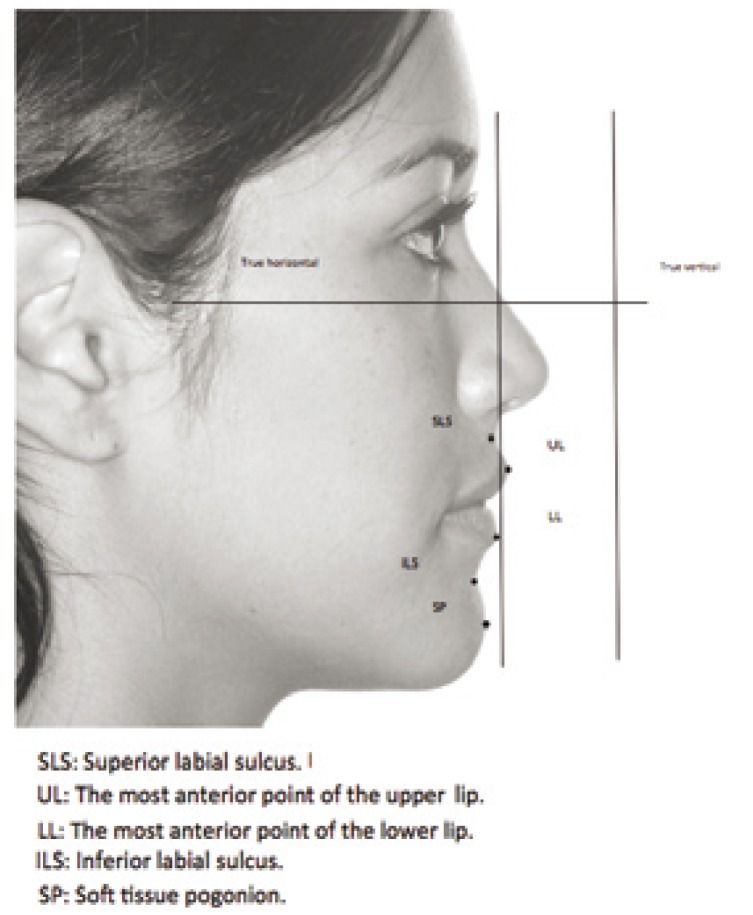
Method and soft tissue points in measuring anteroposterior profile to the subnasale vertical perpendicular to the true horizontal (Spradley 18 1981).

Spradley (10) published the means, standard deviations and ranges of the anteroposterior positions of five points in the soft tissue below the nose in young adults with aesthetic profiles and normal sagittal and vertical skeletal relationships (with ANB angle, depth of the maxilla, facial axis, wits and vertical values within the normal reference ranges). This author used linear measurements from four different reference planes, i.e., subnasale vertical perpendicular to true horizontal and to the horizontal Frankfort plane, and nasion vertical perpendicular to true horizontal and to the Frankfort plane. After tracing the True Horizontal (TH) (using a radiopaque grid) and True Vertical (TV) lines, a perpendicular was traced to TH passing through the subnasale, defining a plane referred to as “subnasale vertical” (Fig. 1). The subnasale is found by bisecting the angle formed by the nasal columella and the upper lip coverage. From this subnasale vertical, measurements were made from the following points: the superior labial sulcus (SLS), the most anterior point of the upper lip (UL), the most anterior point of the lower lip (LL), the inferior labial sulcus (ILS), and the soft tissue pogonion (SP). It should be noted that these measures are not dependent upon the position of the chin.

The use of the vertical perpendicular to the horizontal of the subnasale point was shown to yield the smallest standard deviation of the four different reference planes, and can be used for the diagnosis of dentofacial deformities in the sagittal plane and for corrective treatment planning. In any case, the planning of orthognathic surgery has been, and remains, the subject of debate.

Assessment of the aesthetic parameters of the facial profile in orthognathic surgery is of great importance for establishing an appropriate treatment plan. Many parameters have been proposed in this sense. The present study was designed to identify the most effective parameters, and to establish which of the most widely used parameters are of an objective nature, with a view to offering an assessment of aesthetics independently of the observer. Such information is necessary in order to establish an objective assessment of the expected aesthetic modifications, and to define which parameter or parameters are most precise in evaluating the facial profile.

Accordingly, this retrospective study aims to offer clinicians a definition of those measures which should be considered from the start, in order to ensure harmony as perceived by any observer. Our study has been made using subnasale line as a reference line, looking to validate it. The Subnasale True Vertical has been used for surgical planning for years. Our aim is to validate the effectiveness of the True Vertical Line (TVL) for this purpose and to verify whether clinical standards was were originally proposed are still valid according to current aesthetic criteria. We used surgical operated patients profile, without any change by morphing or software (as the most of articles previously published).

## Material and Methods

For the testing of our hypothesis, we used a sample of subjects divided into three groups: a first control group of normocclusives subjects; a second group of Class II surgical patients at a time previous to surgery, and a third group of the same Group 2 patients after undergoing orthognathic surgery and have normalized their occlusion and facial features.

A total of 52 individuals were evaluated, divided into this three different groups: (a) Group 1 is our control group, consisting in profile silhouettes of 20 individuals with the following characteristics: subjects with normal occlusion; aged ≥ 18 years; absence of previous orthodontic treatment; bilateral molar and canine Class I; positive or negative dental bone discrepancy no greater than 2 mm, with no transverse malocclusion, an overjet of between 1 and 3 mm, and an overbite of between 2 to 4 mm; posterior and anterior rotations not exceeding 15º, in more than two teeth of the anterior zone.

The second and third groups each consisted of 16 patients; (b) Group 2 consisted of 16 subjects (11 women and 5 men) aged between 23 and 46 years, with severe Class II malocclusions. These patients all needed skeletal correction through orthognathic surgery. Furthermore, as a determining factor, we considered patients with a facial convexity angle (11) of less than 165º (mean facial convexity 159.99º). Arnett and Bergman (12) reported that the facial convexity angle is specific for determining the severity of the skeletal class, and is even able to define the need for orthognathic treatment in cases with severe deviations (standard Class I between 165º and 175º, Class II < 165º, and Class III > 175º). The aim of treatment is to correct the skeletal and dental anomalies inherent to normalization of the parameters. Some of the patients had undergone extractions to reduce crowding, or to produce the overjet needed with surgical correction; (c) Group 3 consisted of the same 16 patients as in group 2, but after the completion of orthognathic surgery. In this case, the type of treatment was not taken into consideration (single maxillary surgery, bimaxillary surgery, mentoplasty), and the diagnosis was taken to ensure the correction needed to solve the anomaly, based on the individual characteristics of each patient.

The group 1 (normal occlusion) presented a mean age of 28.6 years, while the age of the patients in the other two groups ranged between 23-46 years (mean 31 years). The orthodontic treatment period prior to orthognathic surgery lasted 17.6 months on average, with a postsurgery treatment duration of 6.2 months – the male/female ratio being 5/11.

This study followed the Declaration of Helsinki on medical protocol and ethics standards being as all subjects which participated in our study were informed and gave their consent previously to their inclusion in it. Due to the nature of this study, all subjects’ profile were digitalized, so there was not possible their personal identification by observers. All participants, subjects sample and observers, were informed about the characteristics of the study and all of they agreed with their participation.

Profiles of all patients were obtained through photographs and lateral X-rays taken in the natural head position (NHP) (13,14) and in centric relationship (CR) (15). Normocclusive subjets’ profiles were obtained trought lateral photographs. They belong to a database of the normocclusive subjects. The profiles were processed with Adobe Photoshop CSE Extended ® version 10.0 for Mac in order to obtain black silhouettes. The profiles were drawn from the upper tissue to glabella G’, and from the lower tissue to cervical point, C’ (intersection of the horizontal and vertical of the neck), with their vertical prolongations. Profile conversion to black silhouettes was carried out in order to discard subjective aspects and facial structure qualities unrelated to the objective measures and proportions (hair and eye color, etc.). Only these black silhouettes on white background were shown to observers, so that no subject of the sample may be facially recognized. Each black silhouette in the entire sample received a random number for identification, positioning in the presentation (PP), and subsequent analysis of the results. All X-rays and profile photographs were made with the same orientation (NHP).

**Figure 2:**
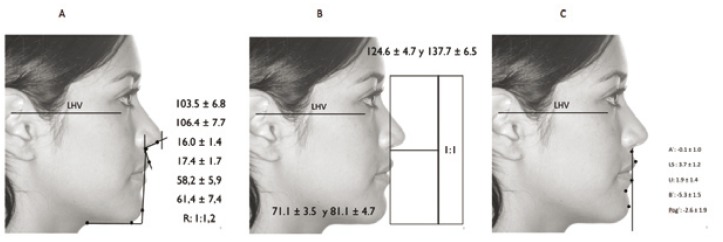
Graphic representation of the study profile measurements. A. Angular measures and relationships. B. Vertical measures. C. Subnasale vertical sagittal measures.

On the other hand, Nemoceph 3.0 Nemotec ® (Software Dental Studio) was used for profile teleradiography (soft parts) cephalometric study of those cases that could be interpreted exclusively from the contours, including the following parameters (16):

Facial profile measurements (Fig. 2):

- Sagittal to the subnasale vertical (true vertical):

Point A (A’), Upper lip (UL), Lower lip (LL), Point B (B’), Pogonion (Pg’)

- Vertical:

Total facial height (TFH), Inferior facial height (IFH), Gap 

- Lines and angles:

Nasolabial angle (NLA), Nasal projection (NP), Neck length (TL) (IFH/TL ratio).

 Table 1 shows each of the measurements and their mean values, for males and females. Likewise, Fig. 2 shows the measures applied to each of the facial profile parameters.

**Table 1:**
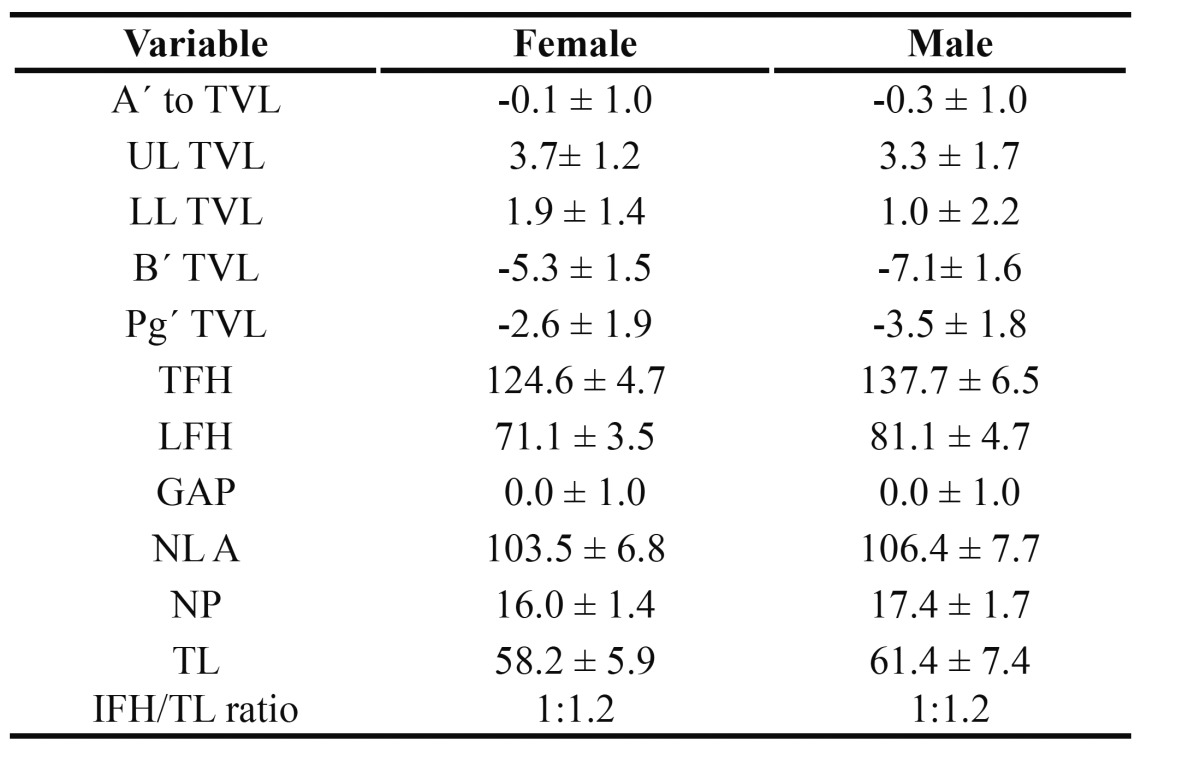
Mean values of the profile measures used in the study, for males and females (Arnett 1999, (27)).

**Figure 3:**
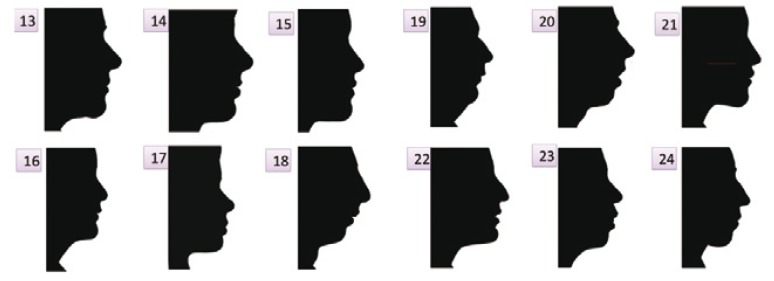
Image presentation on two screens. Six black silhouette profiles were placed on each screen.

All profiles were integrated as a Microsoft Power Point presentation (PP) (Fig. 3), with 6 profiles on each of two screens. The subjects were allowed 20 seconds of observation, after which they wrote down the measurements and characteristics as reflected in the questionnaire.

In order to ensure reliable and reproducible results, all images presented numbering visible to the observer, but without any reference to either the patient or the corresponding group. Likewise, none of the observers knew the nature of the subjects, the fact that there was a control group, or that groups 2 and 3 involved the same patients, but in two different evolutive stages of treatment.

Each presentation, with all the profiles of the three groups, was presented independently to three types of observers (n=72):

● Subjects not trained in the assessment of facial aesthetics (n=24): 7 males and 17 females, with a mean age of 25.2 years (range 20-28).

● Maxillofacial surgeons with usual practice in orthognathic surgery and different levels of experience (n=24): 9 males and 15 females, with a mean age of 39,6 years (range 25-55).

● Orthodontists, all with theoretical and practical knowledge of facial planning in patients requiring orthognathic surgery for malocclusions (n=24): 18 males and 6 females, with a mean age of 38.2 years (range 26-56).

The first 6 images were not taken into account, and did not correspond to the mentioned groups; they were used to familiarize the observer with assessment of the required data (17), and corresponded to patients randomly selected from the orthodontics clinic.

The mentioned questionnaire in turn addressed the following aspects : Aesthetic assessment based on a 5-point Likert scale (18,19) (1=very poor profile, 2=poor profile, 3=good profile, 4=very good profile, 5=excellent profile). No additional information referred to the procedure was specified.

Intra-observer concordance was checked with the kappa index, repeating the test among the observers 30 days after the first evaluation.

- Statistical analysis: Calculation of the sample size has been made using two previous articles (20,21), where the observations analyzed were 2960 and 2100 respectively. In our study, the total amount of observations reached 3744.

Furthermore, we calculated the size of each group based on a pilot study in which, through the following formula was obtained a sample size of 10.49 subjects.

n = 2(Zα+Zβ)2 S2d / Xd2

n = 2 (1,96 + 0,842)2 0,522 / 0,781 = 10,49

Intra-observer correlation was used to evaluate the perception sensitivity of the method used, and to discard randomness in assessment (kappa index).

Previously, we performed an analysis of the normal distribution of the sample. After verifying this, a simple blind study design was made to evaluate the cephalometric results and the aesthetics assessment of the profiles. An Anova and a T-student tests were made to assess pre-/post- significance. It was used the SPSS version 18 statistical package. In all cases statistical significance was considered for p<0.05.

## Results

The intra-observer correlation index (Kappa index) ranged from 0.531 to 0.828, indicative of moderate good to very good results. These values confirm the reliability and reproducibility of the data obtained from the different observers.

Comparison of the aesthetic assessment data ( Table 2) among the three groups of observers did not show statistically significant results within the same patient groups (for example, the mean scores for group 1 were 2.50 ≈ 2.65 ≈ 2.55). On the other hand, as can be seen in the table, the orthodontists evaluated with a better score than the other two observer groups (laypeople and maxillofacial surgeons).

**Table 2:**
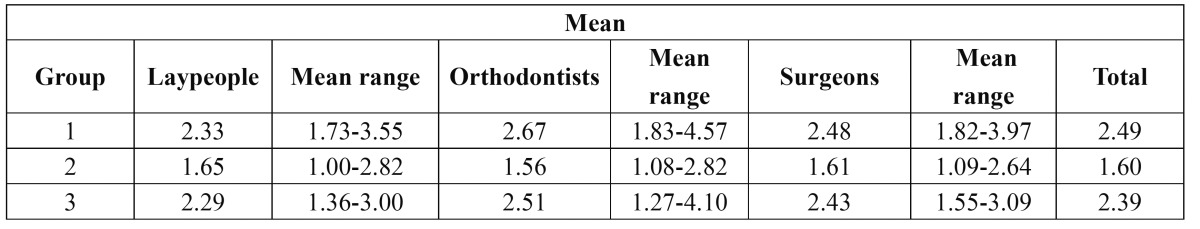
Aesthetic assessment parameters of the three observer groups.

The mean aesthetic assessment scores were: Group 1 (control / normal occlusion): 2.5699 (range 1.97-3.97, SD 0.528); Group 2 (Class II, presurgery): 1.6647 (range 1.04-2.66, SD 0.471); and Group 3 (Class II, postsurgery): 2.4595 (range 1.46-3.35, SD 0.594). These results indicate a clear increase in aesthetic perception after orthog-nathic surgery, with scores similar to those recorded in the control group (subjects with normal occlusion). Significant differences (p<0.001) were observed on comparing the mean aesthetic assessment scores between the normocclusive group and group 2. However, it was not observed between the normocclusive group and group 3 ( Table 3,  Table 4).

**Table 3:**
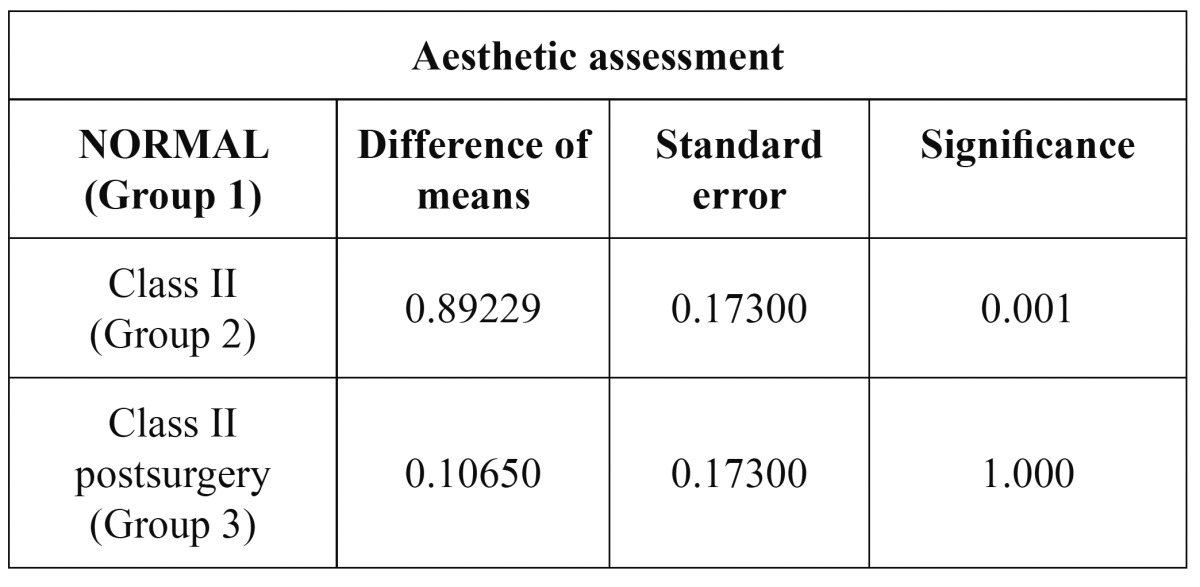
Statistical significance between the three groups of patients studied.

**Table 4:**
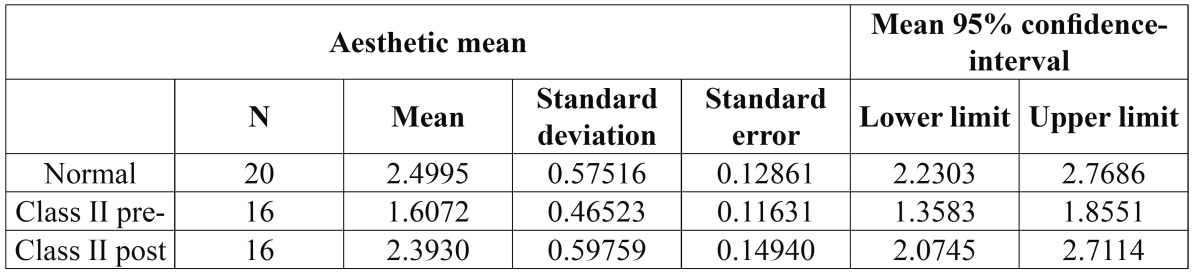
Aesthetic assessment parameters of the three observer groups.

Considering each factor independently, the parameters soft tissue point B (B’) and the soft tissue pogonion (Pg’) were found to be significantly different in groups 2 and 3. A positive correlation was observed, since the inferior third was modified, together with projection of the mandibular structure (B’ and Pg’), following orthognathic surgical correction of Class II malocclusion (group 3).

Likewise, GAP, a facial profile factor, proved quite different for the three groups, with mean values of 1.315 in group 1 (normal occlusion controls), 5.819 in group 2 (Class II presurgery patients) and 3.094 in group 3 (Class II postsurgery patients) – the differences being statistically significant (p<0.000). In turn, IFH showed decreasing values in the following order: group 2 (77.306) > group 3 (71.225) > group 1 (68.100), though in this case statistical significance was not reached. Point B´, in the same way as IFH, decreased in the same order, with values of 11.544, -7.856 and -5.775 in groups 2, 3 and 1, respectively (p < 0.001). These values indicate that surgical correction (group 3) results in a patient facial profile close to normal. Another parameter crucial in Class II alterations and in subsequent corrective treatment is the pogonion (Pg’). The controls with normal occlusion (group 1) presented a mean Pg’ with respect to the subnasale vertical of -3.660; while the group 2 patients (Class II presurgery) showed a mean value of -10.569. However, following the correction of bone deformation (group 3), the Pg’ values improved significantly, approaching the values recorded in the control group. It should be noted that the value -4.606 in group 3 (Class II patients after orthognathic surgery) is very close to the value -3.660 recorded in the control group. Furthermore, there were significant differences between groups: group 1 versus group 3 (p<0.001) and group 2 versus group 3 (p<0.008, i.e., lesser significance).

## Discussion

Intra-observer concordance testing with the kappa index revealed high sensitivity in the aesthetic assessment of the sample (0.531 and 0.828,), in coincidence with similar studies using this same system and in which values close to our own were recorded (between 0.46 and 0.78) (22,23).

The present study involved an aesthetic assessment of the observed patient profiles in which the findings in group 1 (controls with normal occlusion) were significantly different from those in group 2 (Class II malocclu-sion prior to corrective orthognathic surgery). The observations coincided with those published by Dunlevy et al. (24), who found increased changes in mandibular position after surgery to be correlated to improved aesthetics. Thus, as was seen in our series, recovered aesthetics can be expected, with scores similar to those recorded among the controls, following the pertinent corrections to harmonize the facial structure after orthognathic surgery. In our study, no improvement in aesthetics was observed in only two cases after treatment. In the first case, the untrained observers yielded a negative assessment, while the other two observer groups yielded a slightly more favorable evaluation. It should be noted that in this case the initial (presurgical) aesthetic assessment was high; as a result, the change in score following treatment may have been less favorable than expected. In contrast, the second case may have been conditioned by the severe initial facial involvement, which was not fully corrected as a result of surgery, due to the important skeletal alterations. We only conducted an aesthetic evaluation of the facial profile, though all of the patients showed very significant improvement of their malocclusion. The existence of a carefully selected control group afforded important information not found in other studies such as those published by Tsang et al. (18) and Shelly et al. (17). These authors only evaluated the relationships before and after orthognathic surgery in Class II patients. In our case, the control group afforded reliable and reproducible information, since the values recorded for the patients after surgery (group 3) were highly coincident with the reference values of the controls with normal occlusion (group 1).

Profitt (25), using color images, reported a correlation between profile improvement and surgical treatment. In our study, and coinciding with the work of Shelly et al. (17), use was made of black silhouettes, thereby avoiding subjective perception bias (eyes, hair, skin, etc.). Arnett et al. (16), in a series of 46 models, only studied facial structure, but not quality (hair, eyes, skin, etc.). This author also established a dividing line between previous and current facial planning, based on the natural position of the head (26) – his findings corroborating the observations of Ludstron and Ludstron (13), with great reproducibility of these registries beyond the use of the Frankfort plane. It is here where the author introduced the difference with respect to the previous studies (14), using the true vertical as the axis for facial planning of the patients in assessing the profiles. This evaluation parameter was first introduced by Spradley (10) as a reference for skeletal class classification. The values obtained with the subnasale vertical perpendicular to the true horizontal showed the smallest standard deviations versus the other commonly used methods. In addition, these values were not dependent upon chin position (10). In his first work, Arnett et al. (12) used the line joining the chin and subnasale originally defined by Burstone (7).

Tsang et al. (18) postulated a reference for diagnosis and aesthetic evaluation of the changes in patient facial profile using the angle of the partial profile, and defined an anatomically conditioned position of the head. This is in contradiction to our own study, since the reference for assessing improvements in the facial profile of skeletal Class II patients requiring surgical treatment is exclusively based on the chin (highly variable).

Arnett et al. (16) described a series of mean aesthetic reference values for facial planning, using the subnasale vertical and the sagittal projections of the lower third. It may be thought that these measures are only applicable to certain population groups, and are not of an objective nature. In this context, the values ideally should be identifiable as being optimum by any observer. In our study we established the high sensitivity of the measures, since by using the control group as reference we were able to detect significant changes between patients before and after corrective surgery. If the system used to assess the sagittal position of the structures of the lower third of the face is efficient, we could expect to obtain alterations in the position of these structures, which are normally affected. Accordingly, in group 2 (Class II patients before surgical treatment) we observed an alteration of the parameters determining the position of the mandible and chin, which must be corrected after surgery. The results of the lower lip, point B’ and pogonion (Pg’), and the vertical dimension, yielded significant differences with respect to the corrected group (group 3) and the reference group of controls with normal occlusion (group 1).

Orthodontists were found to be more precise in assessing the facial profile results, determining the degree of harmony and beauty of the facial profiles, according with other studies (26-28). In our series the observers yielded similar mean values in assessing profile aesthetics. There were no significant differences in profile assessment among the different observers within one same group. In contrast, and in coincidence with the findings of Naini et al. (29) and Tufekci et al. (30), there were evident differences in the appreciation of facial profiles between different observer types or groups. The mentioned authors described dental professionals as being conditioned by their training, tending to be too critical of any deviation from the norm. In our study we compared the opinion of orthodontists, maxillofacial surgeons and untrained observers (undergraduate dentistry students) – the most precise results corresponding to the group of professionals with knowledge in the field. These observers were able to distinguish features coincident with the norms of facial aesthetics from those parameters in conflict with such norms - with more precise evaluation of each of the profiles than the untrained observer group. The latter showed lesser criterion in assessing the profiles, assigning clearly lower scores than the orthodontists and maxillofacial surgeons in both the presurgical profiles (group 2) and in the patients who had already undergone orthognathic surgery and complied with the clinical norms (group 3). Nevertheless, the operated patients still obtained higher scores from the untrained observers than the presurgical patients. In coincidence with our observations, Tsang et al. (18) found specialists to be more critical, with better perception of the changes between final outcome versus baseline, than the general population.

So that, in our study we concluded that significant differences were observed in aesthetic assessment between subjects with normal occlusion (group 1) and presurgical patients with Class II malocclusion (group 2). The least aesthetic profiles in the series corresponded to the latter group, while aesthetic assessment in group 1 was seen to be similar to that of the postsurgical Class II patients (group 3).

As expected, the cephalometric values (point B’, and pogonion) received a more positive evaluation in the postsurgical and normal occlusion profiles – the poorest values corresponding to group 2 (Class II prior to orthognathic surgery).

The subnasale vertical and sagittal measurements of the lower third of the face are crucial to facial aesthetics, and therefore for planning orthognathic surgery. Consequently, these aesthetic parameters can be used in ortho-dontic treatment planning according the current aesthetic criteria.
